# Development and Validation of Two Questionnaires to Study the Perception of Conflict in Physical Education

**DOI:** 10.3390/ijerph17176241

**Published:** 2020-08-27

**Authors:** Unai Sáez de Ocáriz, Pere Lavega-Burgués

**Affiliations:** 1 Motor Action Research Group (GIAM), INDEST, National Institute of Physical Education of Catalonia (INEFC), University of Barcelona, 08038 Barcelona, Spain; 2 Motor Action Research Group (GIAM), INDEST, National Institute of Physical Education of Catalonia (INEFC), University of Lleida, 25192 Lleida, Spain; plavega@inefc.udl.cat

**Keywords:** adolescents, measurement, motor conduct, motor praxeology, peace education, psychometric properties, psychosocial aspects, physical activity, school life, well-being

## Abstract

Improving the quality of teaching and learning, as well as school coexistence are international priorities for the new educational challenges of the 21st century (UNESCO 37 C/4 resolution). Physical Education (PE) has become a key subject for education on school coexistence by enabling significant motor experiences to promote interpersonal relationships and transform motor conflicts (MC). The objective of this research was to develop and validate two questionnaires (CONFLICT1-AGE and CONFLICT1-RES) to study secondary school students’ perception about MC in PE. Study 1 searched for evidence related to their content validity and response process validity, and Study 2 examined internal structure, reliability, and concurrent validity. As a result, a seven-item single-factor model was selected for CONFLICT1-AGE, and a five-item single-factor structure was chosen for CONFLICT1-RES. Both models exhibited an excellent fit to the data, where CONFLICT1-AGE: χ^2^ (*df*) = 18.621 (14), *p* = 0.180, RMSEA (90% CI) = 0.033 0(0.000–0.069), CFI = 0.994, TLI = 0.991; CONFLICT1-RES: χ^2^ (*df*) = 13.350 (5), *p* = 0.020, RMSEA (90% CI) = 0.075 (0.027–0.125), CFI = 0.986, TLI = 0.972. Furthermore, both questionnaires presented satisfactory internal consistency (α_CONFLICT1-AGE_ = 0.745, α_CONFLICT1-RES_ = 0.737). Their combination will provide a wide view of secondary school students’ perception about MC.

## 1. Introduction

This article provides two questionnaires on conflicts for the Physical Education (PE) teacher, which is of high interest in the current education system. Indeed, optimizing the quality of education and school coexistence are important objectives at an international level to address the challenges of the 21st century towards more fair and participatory societies. Schools are transformed into spaces for the construction of democratic, peaceful, and inclusive learning processes that promote community coexistence (resolution 37 C/4) [1]. Understanding this social reality and proposing effective and creative teaching strategies are the current educational challenges. As UNESCO [1] confirms, promoting peaceful societies of respect, gender equity, diversity, sustainability, and active participation in democratic processes through critical reflection, creativity, and responsibility are core pedagogical aims.

Coexistence as a basis of education is not new. At the “International Commission on Education for the 21st Century”, Delors [2] already described that coexistence was one of the foundations for 21st century education. He confirmed that educational processes that are generated in schools contribute to develop social skills based on respect for diversity and peaceful strategies as a way to resolve conflict situations. “Learning to live together”, one of the “four pillars of education” [2], is still one of the most important challenges for 21st century schools [3,4].

In the last decade, educational objectives have focused on constructing positive school climates that favour the development of learning acquisition and students’ integral growth in a peaceful environment of coexistence that creates democratic awareness, in the context of mutual respect [5,6,7,8].

The influence of current socio-economic, cultural, and educational transformations requires schools to not only promote the academic skills of their students, but also to encourage social skills and relational and emotional well-being as the basis for optimal development of school-age children [9,10,11,12,13]. This is confirmed by different studies [14,15,16,17], underlining the importance of school children’s current stage of maturation.

School is an environment in which boys and girls spend a large amount of time and where several meaningful experiences are generated. It is a critical context for their present and future socio-emotional and behavioral adjustments [3,18,19,20]. In fact, between 11 and 16 years old, which is a transition stage marked by intense physiological, physical, psychological, and social changes and transformations [5,20,21], school becomes a relevant scenario for students’ integral development because of: (a) impacts on coexistence and well-being [4,22], (b) social commitment [13], and (c) transfer into the school community [23,24].

In practice, school coexistence presents difficulties that disturb the school climate [6,25,26]—a fact that causes interpersonal conflicts’ emergence [27,28]. This reality opens a path towards education on interpersonal relationships in favour of growth in social competencies that help students with constructive conflict transformation [23,29,30].

### PE, Motor Conduct Education, and Conflict 

At schools, physical activity and sports are an exceptional way for students to experience interactions with their peer group [31] and to develop social and communication skills, pro-social behaviors, and respect for others [32,33]. As a consequence of that interaction, boys and girls shape their cognitive, emotional, and social development [34], and build and optimize their values scale and moral thinking [35,36,37].

As UNESCO affirms, PE represents a learning input to develop necessary skills to be successful in the 21st century, because it is the only curricular subject whose approach combines motor skills and value-based learning [38]. This was also confirmed in the Berlin declaration (2013) at the UNESCO International Conference of Sports Ministers (MINESP V), where it was stated that PE is the most effective way for children to have competences, skills, and attitudes to participate in society throughout life [39].

According to the International Council of Sports Sciences and Physical Education [40], PE helps students to develop respect for themselves and others in a comprehensive way, improve self-confidence and self-esteem positively, optimize social-cognitive-academic performance, interact with others and learn social skills, and be able to have experiences towards well-being. This knowledge will transfer to the rest of their life in helping to improve social and school coexistence [41,42].

In PE, an area based purely on procedural experiences, the teaching–learning processes are the consequence of motor conduct acquisition and transformation [43]. Each motor game has an internal logic that requires the players to interact in a certain way with the rest of the participants—with the space, with time, and with the material [44]. As a result of these interactions, each student adapts their responses to the internal logic of the game through different motor conducts.

The motor conduct concept determines that any motor response (e.g., “a partner pass”) mobilizes his/her global personality, activating organic (physiological), affective (emotional reactions), cognitive (decision-making), and social (type of relationships with other players) dimensions [43]. Interpreting PE as motor conduct education provides teachers the possibility of integrating significant experiences in the comprehensive training of their students [44,45,46].

Educating motor conducts means transforming conflictive student interventions in PE classes to improve school coexistence [47]. In the education of interpersonal conflicts, traditional games are a fundamental element to promote prosocial learning through significant experiences of relational well-being [23,28,29,43,48].

In any traditional game, players must solve different problems related to the internal logic of this activity, presenting different relational challenges. For this reason, any traditional game is a real social interaction laboratory [43]. When motor conducts are adequate (adjusted), coexistence develops in a favourable relationship environment. Nevertheless, when disagreements, imbalances, or tensions occasionally emerge in relationships, motor conduct that do not favour interpersonal relationships are generated [47].

Consequently, a motor conflict (MC) can be produced by misadjusted verbal agreement conduct (when establishing agreements with the other participants), misadjusted motor conduct (if deviating from the expected responses), or by perverse motor conduct (which generates disorder and that implies breaking the game’s rules). Subsequently, in response to this stimulus, involved players may react by verbal (e.g., an insult), physical (e.g., a push), or mixed (e.g., insult plus push) aggression [49,50]. After observing these MCs, PE teachers need strategies and instruments that help them to educationally transform situations of imbalance, and allow themselves to propose positive experiences that favour the integral development of their students [49,51,52].

In this sense, Sáez de Ocáriz and Lavega [47] introduced the conflict index (ICF) as a key element for MC education, which allows to quantify the conflictive intensity level of the protagonists to later propose, design, and put into practice coherent strategies for the improvement of their conflictive conduct [49]. The ICF is the result of the sum of the intensity of the origin, where three possibilities of conflictive conducts are considered (agreement = 1; misaligned = 2; perverse = 3), with the intensity of the conflictive response, by three possibilities (verbal aggression = 1; physical aggression = 2; mixed aggression = 3). In both cases, the scores range from 1 to 3 depending on their intensity level, with 1 being the least intense and 3 the most intense [47].

After obtaining a conflictive profile (ICF), a teacher has the possibility of introducing preventive measures to promote positive dialogue and relational well-being among his/her students [30,49]. Accordingly, a teacher can intervene fundamentally: (a) Through the motor game’s internal logic, by modifying the rules, or even changing the game to look for more favourable conditions; and (b) with regard to the conflictive situation’s protagonists, where according to the ICF, a teacher will be able to directly intervene or provide time and space to the involved actors in order for them to manage the conflict in a positive way [29,49].

Consequently, MC education becomes a fundamental pedagogical strategy for the PE teacher. Teaching–learning processes will be optimized through MC transformation, and will favor the improvement of school coexistence [23,29,46,53,54]. Furthermore, if the teacher is able to involve students in their own teaching–learning process by reflecting on the lived processes, it is possible to incite the appearance of social and individual transformations that bring significant improvements to school coexistence [55].

It is necessary to have strategies and resources to promote peaceful school coexistence environments [5,6,7,8]. Introducing strategies to encourage the student to become aware of and reflect on their intervention and progress in the knowledge they have achieved could foster meaningful learning. For this reason, it is appropriate to go deeper in the perception of students as a tool to develop critical reflection and responsibility in schools [1], especially in the PE area. Developed experiences that facilitate participants’ relational well-being is a key element to optimize interpersonal relationships and a positive climate in the classroom [35].

After reviewing the literature, we detected the existence of some examples of questionnaires close to the study’s objectives, with a general orientation towards the (a) classroom climate (e.g., “Spanish version of Sport Motivation Scale II adapted to physical education -SMS-II-PE” [56]; “Learning Climate Questionnaire-LCQ” [57]; “Physical education classroom management instrument” [58]), (b) school violence (e.g., “School Violence Questionnaire-CUVE” [59]; “Test AVE—Bullying and School Violence” [60]), (c) school coexistence (e.g., “School Coexistence Scale” [61]; “Non-violence questionnaire—CENVI” [62]), (d) conflict resolution at school age (e.g., “Conflictalk Spanish version” [63]), (e) physical activity (e.g., “Physical Activity Questionnaire for Older Children—PAQ-C” [64]), and (f) PE (e.g., “Prosocial and Antisocial Behavior in Sport Scale to the Spanish context of PE classes” [65]; “Appropriate Conduct Questionnaire in Physical Education and Sport -CAEFD” [66]), among others.

These orientations were considered inadequate for the study of conflicts in PE, as they did not focus on the elements that are part of the MC process (i.e., MC causes and their responses) [50]. Getting students’ opinions and their point of view about MC, responses derived from the previous conflicting stimulus, and even both at the same time could help PE teachers to complement the MC observations that emerge in their classes. This action would allow the teachers to make decisions and make coherent proposals aimed at optimizing motor conduct education of their students, and consequently, promote school coexistence towards relational well-being and the creation of positive environments.

Under the theoretical perspective developed by the MC [49], the objective of this research was to develop and validate two questionnaires to study secondary school students’ perception about MC in PE lessons. The first questionnaire (CONFLICT1-AGE) explored the perception of the students about the different possibilities that an MC can cause, while the second (CONFLICT1-RES) examined students’ perception of the responses to the provocative stimuli from MC. The combination of both instruments would allow a closer view of the secondary school student’s perception of MC.

The first questionnaire allows us to identify how the involved students have adapted their responses to the internal logic of the game. When participants do not adapt correctly to this internal logic, it could cause tension in the interpersonal relationship. However, it may be that the perception of the student receiving the conflict has not been absolutely adequate. Therefore, it is necessary to focus the attention first on the origin of the conflict. The second questionnaire deals with the reaction to a conflictive relationship and places us in the context of the regulation of negative emotions and the attitude adopted in a conflict. In this case, the intervention of the PE teacher must be different from that of the origin of the conflict. Hence, there is a need to consider the two questionnaires as an inseparable binomial in order to understand the two dimensions of the MC process.

## 2. Study 1. Item Development, Content Validity, and Validity Based on Response Processes of CONFLICT1-AGE and CONFLICT1-RES

The purpose of Study 1 was to develop a battery of possible items for the CONFLICT1-AGE and the CONFLICT1-RES, and then obtain evidence related to their content validity and validity based on response processes [67].

Following the suggestions of Boateng et al. [68], different strategies were used to develop and select the most appropriate items, with both deductive (i.e., review of the theoretical framework) and inductive methods (i.e., researchers’ discussions), to assess that the items adequately measured the domain of interest (i.e., panel of experts) and to ensure the items’ comprehensibility (i.e., focus groups with members of the target population).

### 2.1. Method

#### 2.1.1. Participants

A total of 15 researchers in the field (10 males and 5 females: *M_age_* = 44.8 years; *SD* = 10.8; range = 26–62 years) participated in two panels of experts. In the first panel, 10 experts with different profiles participated: Senior Lecturers with research experience (*n* = 4), a PhD with extensive teaching and research experience (*n* = 1), two PhDs with novel teaching experience (*n* = 2), and a PhD student (*n* = 3). In the second panel were five external experts in the field with teaching and research experience at university level, Senior Lecturers who specialized in the subject (*n* = 2), in methodology (*n* = 2), and at formal education level (*n* = 1).

Subsequently, 16 high school students participated in the focus group (*n* = 8 boys and 8 girls; *M_age_* = 14.1 years; *SD* = 1.5; range = 12–17 years), distributed into the 1st year (*n* = 4; 2 boys and 2 girls), 2nd year (*n* = 4; 2 boys and 2 girls), 3rd year (*n* = 4; 2 boys and 2 girls), and 4th year (*n* = 4; 2 boys and 2 girls).

#### 2.1.2. Procedure

**Figure 1:**
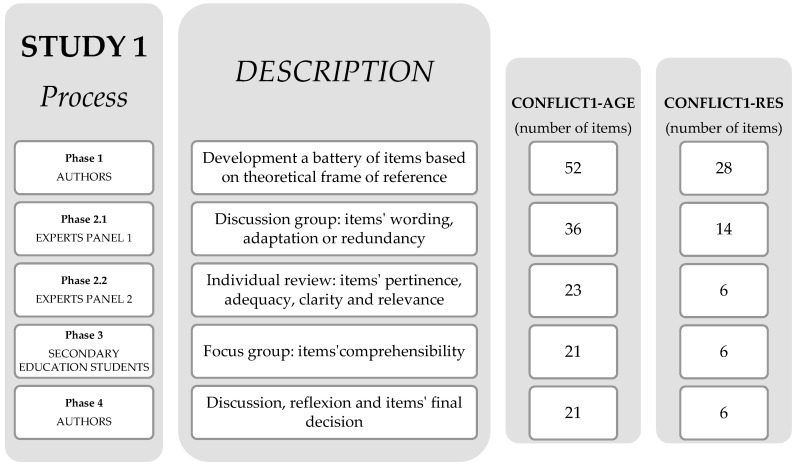
Study 1 process description.

The requirements outlined by the Clinical Research Ethics Committee of the Sports Administration of Catalonia were followed (certificate with reference number 05/2019/CEICEGC). Study 1 is based on four phases (Figure 1). The development of both questionnaires followed a similar procedure and aimed to create two instruments to study the perception of students’ motor conflict in the Secondary Education PE lessons [47]. The items reflected the fundamental concepts of the two basic elements of the motor conflict: the agent that generates it and the response from the reaction to the previous conflictive stimulus [49]. 

In Phase 1, authors developed an initial pool of items for each questionnaire based on the theoretical framework of reference [49]. To ensure the quality of the questionnaire, four expert compilers were recruited. These researchers had more than 10 years of training in motor praxeology, emotional education, physical education in secondary school expertise, and conflictology. They developed an initial list of items referring to the relationship between the internal logic of games and the different ways in which a diversity of motor conflicts may originate, as well as the response to these previous conflictive stimuli (MC theoretical frame key elements). Specifically, four criteria were followed to develop the initial pool of items—three theory-driven criteria (i.e., items referred to the internal logic of the game, to the MC generating agent and to the conflictive response to the previous stimulus) and one related to the understanding of the items (i.e., wording adapted to the adolescent population). It has to be noted that the items were originally developed in Spanish, but for the article’s preparation they were translated into English (both Spanish and English items are available from the corresponding author upon request). Four researchers participated in this action through a process of translation and back-translation. The items were translated into English individually, and afterwards a definitive version was agreed. Finally, the items were translated back into Spanish to confirm the validity of the translation.

In Phase 2, we teamed up with two expert panels. In the first panel, the discussion group was used as a strategy to review and select the initial pool of items, focusing on the wording, meaning, or redundancy of the items. Approximately 10 days before the meetings, the necessary information was sent to the experts to explain the item development process. The documentation consisted of a text defining the objective of the questionnaires, the theoretical framework of reference, the structure followed by the authors for their design, and the proposal of the initial items. The discussion group met in two sessions of 1.5 h in consecutive weeks (3 h), and the selection of items was agreed for the following verification.

In the second panel, the experts individually conducted an independent review of each item, and provided written comments on their pertinence, adequacy, clarity, and relevance. They received the same documentation as the Panel 1 discussion group, the protocol to validate each item, and the evaluation sheet with a list of the items selected by the first panel. To carry out the validation, the experts had approximately 40 days before the return of the report, and the documentation was sent in different formats (Word, PDF, Excel) to facilitate the researchers’ work. Subsequently, the authors analysed the data provided by the experts, reviewed their contributions and suggestions, and decided to confirm the final items for the two questionnaires in the next phase.

In Phase 3, we conducted two focus groups to assess the understanding of the items from the previous phase and to confirm their comprehensibility by the target population. The students were divided into three different groups. In each of them, we balanced gender and academic equality. The meetings were held on different days and for approximately 40 min each. Reduction of inter-group contact was ensured to avoid biased opinions. First, students were informed about the objective of the study and agreed to participate by signing an informed consent form. Subsequently, the students read the questionnaires and indicated any items were not understood. Finally, the students shared their doubts and generated an internal debate in order to confirm the comprehensibility of the items of the two questionnaires. With informed consent, the sessions were audio recorded for later analysis.

In Phase 4, the authors reviewed the entire process to ensure all the fundamental suggestions related to the perception of the agent generating the MC and the response derived from the conflicting stimulus were included [47,49]. At the end of the process, CONFLICT1-AGE—Version 1 and CONFLICT1-RES—Version 1 were developed.

### 2.2. Results and Discussion

#### 2.2.1. CONFLICT1-AGE

In Phase 1 (Figure 1), the authors generated a pool of 52 potential items based on the fundamental concepts of the motor conflict generating agent [49]. In Phase 2, the first panel of experts began working from the 52 final items of Phase 1, and accentuated possible changes in the wording, adaptation, or redundancy of the items in the questionnaire. As a result, 19 items were maintained and 21 were deleted. Among the deleted items, 13 were removed for having similar content (e.g., “In the game, the conflicts I have caused have been so because I’ve been cheated”) and nine items for having an incorrect description that made it difficult to understand (e.g., “The conflicts in which I took part during the game have been cause by not respecting the agreements”). In addition, 11 items were modified to improve their wording (e.g., “In the game, the conflicts I have caused have been so because someone did not follow game’s rules” adapted to “In the game, the conflicts I have caused have been so because someone fooled the game”), and six new items were proposed (e.g., “In the game, the conflicts I have caused have been so because someone fooled the game which affected me”). Therefore, this procedure resulted in 36 items.

Afterwards, the second panel of experts assessed the pertinence, adequacy, clarity, and relevance of the remaining items. As a result, 21 items were retained and 15 items were eliminated: three items seemed to not be obtaining relevant information about the phenomenon under investigation (e.g., “In the game, I’ve always been related to the same person”), four items to not be adequately formulated for high school students (e.g., “In the game, I’ve had conflicts about hurting another participant”), seven items to not be clear enough (e.g., “In the game, I have had conflicts due to the mistakes of other participants”), and one item not be theoretically relevant (e.g., “When I’ve been involved in a conflict in the game, I’ve mostly felt that we both won”). Additionally, the experts proposed two new items (e.g., “In the game, the conflicts I’ve caused have been because I’ve hit someone else”). This phase finished with a total of 23 items.

In Phase 3, students’ focus groups reviewed the 23 items from the previous phase to assess their understanding. Twenty-one items were maintained, and two items were eliminated because they generated high doubts (e.g., “In the game, I have had conflicts for offending another participant”). This procedure resulted in 21 items.

In Phase 4, the authors reviewed the entire process to ensure all the fundamental suggestions from the previous steps had been included [47,49]. It was agreed not to delete or add any item. This process resulted in a total of 21 items, and gave rise to CONFLICT1-AGE—Version 1.

#### 2.2.2. CONFLICT1-RES

For this second questionnaire, the same previous procedure was followed.

In Phase 1, the authors generated a pool of 28 possible items based on the fundamental concepts of the response derived from the conflicting stimulus [49].

In Phase 2, the first panel of experts reviewed the initial 28 items specified in Phase 1, and suggested changes in the wording, adaptation, or redundancy of those items. As a result, 10 items were retained, nine items were removed for having similar content (e.g., “In the game, I have reacted with a hit when another participant has pushed me to the limit”), and five items for having wording that made it difficult to understand (e.g., “When I generated the conflict, the response has mostly been to insult and beat me”). Simultaneously, four items were modified to improve their wording (e.g., “In the game when I caused the conflict, response has mostly been insults” was changed to “In the game, I’ve been insulted when I generated a conflict”). No new items were added in Phase 2. Therefore, this process ended with 14 items.

The second panel of experts validated the pertinence, adequacy, clarity, and relevance of the remaining items. Six items were retained and eight were eliminated: three items for not being adequately formulated for high school students (e.g., “Above all, I reacted with physical aggression when the other participant pushed me to the limit”), and five items because the wording was not sufficiently clear (e.g., “When I have generated the conflict, the response has mostly been annoying me with a physical aggression”). No new items were proposed, and thus a total of six items were retained at the end of this phase.

In Phase 3, students’ focus groups reviewed the six items from the previous phase to assess whether they understood them correctly. All the items were retained without modifications, and no new items were added. Therefore, this procedure resulted in six items.

In Phase 4, the authors reviewed the entire process to ensure all the fundamental suggestions from the previous phases had been taken into consideration [47,49]. It was agreed not to delete or add any item. This process ended in a total of six items, and gave rise to CONFLICT1-RES—Version 1. 

## 3. Study 2. Internal Structure of CONFLICT1-AGE and CONFLICT1-RES

The purpose of Study 2 was to explore and refine the structures of CONFLICT1-AGE—Version 1 and CONFLICT1-RES—Version 1 and to provide validity evidence based on their internal structure. As both questionnaires were expected to be single-factor structures, we used a confirmatory factor analysis approach. In addition, we also assessed the reliability and concurrent validity of both scales.

### 3.1. Method

#### 3.1.1. Participants

Participants were 596 secondary education students (*M_age_* = 14.34, *SD* = 1.59, age range = 12–18 years; 52.7% female). Students were distributed among the different secondary education courses (first year = 17.1%, second year = 24.7, third year = 17.6%, fourth year = 24.3%, fifth year = 16.3%). At the moment of data collection, participants were attending their regular physical education classes.

#### 3.1.2. Instruments

CONFLICT1-AGE: This questionnaire aimed at measuring the perception of the students about the different possibilities that a MC can cause. Participants responded to Version 1 of this questionnaire, which resulted from Study 1. This version included 21 items that were expected to fit in a single factor. Participants were asked to reply to the stem, “In the game, the conflicts I have caused have been so because (…)” using a five-point Likert-type scale with anchors ranging from 1 (totally disagree) to 5 (totally agree).

CONFLICT1-RES: This questionnaire assesses students’ perception of the responses to the provocative stimuli of MC. Students responded to Version 1 of this questionnaire that was developed in Study 1. This measure is a single-factor instrument that comprises six items. To complete the questionnaire, participants were asked to reply to the stem, “In the game, when (…)” via the five-point Likert-type scale described above.

#### 3.1.3. Procedure

We contacted schools and parents of children to get their informed consent. Subsequently, we agreed on dates for sessions of online data collection. Students were informed about the purpose of the study, encouraged to respond honestly, and ensured data confidentiality. Participants, as well as their parents provided informed consent to participate in the study and responded to the CONFLICT1-AGE and CONFLICT1-RES. Sessions of data collection took about 12 min. Despite the fact that researchers were not physically present at the sessions of data collection, PE teachers were directly in contact by telephone to resolve any doubts.

#### 3.1.4. Data Analysis

Data were analysed using SPSS version 17 and Mplus version 7.4 [67]. First, preliminary analyses included the detection of missing values and the analysis of data distribution. Subsequently, the factor structures of the CONFLICT1-AGE and CONFLICT1-RES were examined with Mplus using confirmatory factor analysis (CFA) to test item–factor associations. To that end, data were randomly split into two samples (i.e., Sample A and Sample B, including 298 participants each). In Sample A, we tested a series of CFA models to refine Version 1 of both questionnaires. We started from the initial 21-item measurement model for the CONFLICT1-AGE and from the six-item measurement model for the CONFLICT1-RES, and then we deleted the items that failed to produce sufficient measurement requirements. According to the theoretical framework, all measurement models were expected to fit a single-factor structure. We considered items for deletion when they presented standardized target factor standardized loadings of <0.40. We also inspected modification indices to find problematic items. Finally, we also considered theory-driven criteria (i.e., perception of conflict in physical education) when necessary. All the items shared similar scrutiny and were deleted after we reached consensus. This process resulted in Version 2 of CONFLICT1-AGE and CONFLICT1-RES.

All CFA models tested with Sample A were estimated based on the weighted least squares means and variance adjusted (WLSMV) estimator, which is more suited to the ordered-categorical nature of Likert scales [69]. Different fit indices were used to test the fit of the CFA measurement models to the data: χ^2^ statistic, Comparative Fit Index (CFI [70]), Tucker–Lewis Index (TLI [71]), and root mean square error of approximation (RMSEA [72]), including its 90% confidence intervals (CI). A non-significant χ^2^ value indicates a close fit between the observed and expected model values. The threshold of acceptable fit for the RMSEA is ≤0.08 (for an excellent fit, ≤0.06 [73]). Additionally, CFI and TLI values > 0.95 are considered as indicators of excellent fit [74].

In Sample B, we further tested and refined the internal structure of Version 2 following the same criteria described for Sample A. As a result, we developed the last version of the questionnaires (Version F). Subsequently, to assess the reliability of CONFLICT1-AGE and CONFLICT1-RES, we used McDonald’s [75] coefficient omega (ω), which is computed from the standardized parameter estimates of the model. Finally, concurrent validity of both questionnaires was analysed via disattenuated correlations between their latent factors. To do so, the two latent factors were included in a single measurement model to compute these correlations. Reliability and concurrent validity analyses were also tested with Mplus version 7.4 [69].

### 3.2. Results and Discussion

#### 3.2.1. Preliminary Analysis

As data were obtained via an online questionnaire, no missing values were found. As can be observed in Tables S1 and S2 (Supplementary Materials), most of the items presented floor and/or roof effects. Thus, these results reinforced the use of the WLSMV estimator in subsequent analysis.

#### 3.2.2. Refinement of CONFLICT1-AGE Internal Structure

The initial 21-item CFA model conducted with Sample A showed an insufficient fit to the data, χ^2^ (189) = 717.109, *p* < 0.001, RMSEA (90% CI) = 0.097 (0.089–0.104), CFI = 0.789, TLI = 0.765. Therefore, deletion of some of the items appeared to be necessary (see Table 1). For example, several items (e.g., Item 1, Item 2, Item 3, Item 4, Item 8, Item 11, and Item 19) failed to provide satisfactory factor loading (i.e., ≥0.40). In addition, modification indices suggested the deletion of items: (a) Item 12 and Item 15, (b) Item 5 and Item 6, and (c) Item 9 and Item 20 in subsequent steps of the analysis. The item deletion process with Sample A resulted in an eight-item, single-factor CONFLICT1-AGE, which showed an acceptable fit to the data, χ^2^ (20) = 76.165, *p* < 0.001, RMSEA (90% CI) = 0.097 [0.089–0.104], CFI = 0.944, TLI = 0.922. This structure was considered Version 2 of the questionnaire. Although the model fit was not excellent, we decided to move to Sample B as there were not clear paths for further item deletion (e.g., all factor loadings were >0.40 and no modification indices suggested item deletion).

The eight-item CFA (Version 2) conducted with Sample B showed a promising fit to the data (see Table 1): χ^2^ (20) = 42.855, *p* = 0.002, RMSEA [90% CI] = 0.062 [0.036–0.088], CFI = 0.972, TLI = 0.960. However, Item 10 presented unsatisfactory factor loading (λ = 0.289) and thus was considered for deletion. As a result, the final seven-item single-factor CONFLICT1-AGE showed a perfect fit to the data: χ^2^ (14) = 18.621, *p* = 0.180, RMSEA (90% CI) = 0.033 (0.000–0.069), CFI = 0.994, TLI = 0.991. In addition, factor loadings were satisfactory, as all of them were greater than the 0.40 cut-off value (see Table 2). In this Table, we also present the data distribution of the retained items. The structure that resulted from this process was labelled Version 3.

#### 3.2.3. Refinement of CONFLICT1-RES Internal Structure

The analysis began with the six-item single-factor model being tested in Sample A (see Table 3). This model provided an acceptable fit to the data: χ^2^ (9) = 34.176, *p* < 0.001, RMSEA (90% CI) = 0.097 (0.064–0.132), CFI = 0.974, TLI = 0.957. However, Item 2 presented a factor loading below 0.40 and was considered for deletion. Therefore, the process of refinement resulted in a five-item single-factor CONFLICT1-RES that showed an improved fit to the data: χ^2^ (2) = 30.380, *p* < 0.001, RMSEA (90% CI) = 0.131 (0.088–0.177), CFI = 0.973, TLI = 0.947. Although the fit of this model could be improved by the deletion of Item 5, whose factor loading was slightly above the criterion (i.e., λ = 0.41), we decided to retain it due to theoretical reasons (have all the options confirmed in the MC conceptual framework). This five-item structure was labelled Version 2 and was tested again with Sample B, showing a satisfactory fit to the data: χ^2^ (5) = 13.350, *p* = 0.020, RMSEA (90% CI) = 0.075 (0.027–0.125), CFI = 0.986, TLI = 0.972. All factor loadings were also salient (>0.40; See Table 4). In this Table, we also show the frequencies of response for each of the retained items.

#### 3.2.4. Reliability and Concurrent Validity

Reliability of CONFLICT1-AGE (Version 3) and CONFLICT1-RES (Version 2) was assessed via McDonald’s coefficient omega. Both coefficients were satisfactory: ω_CONFLICT1-AGE_ = 0.756, ω_CONFLICT1-RES_ = 0.757. Additionally, concurrent validity was analysed via a disattenuated correlation between CONFLICT1-AGE (Version 3) and CONFLICT1-RES (Version 2). The correlation between these factors was 0.440 (*p* < 0.001), which supports the idea that both questionnaires pertained to the same theoretical framework. The final version of the two questionnaires is available from the first author about request.

## 4. General Discussion

The purpose of the two studies was to develop and validate two questionnaires to study secondary school students’ perception about MC in PE lessons (i.e., CONFLICT1-AGE and CONFLICT1-RES). Research on conflict education [7,13,28,49] understand conflict as an opportunity. In this sense, the challenge lies in trying to make the students capable of assuming a behavior model based on a “win–win” attitude. For this, it is necessary to focus on the causes that have initiated the CM (origin) and the reaction (response to the previous conflictive stimulus). The findings of the two studies presented could help PE teachers to consider education on interpersonal relationships. In the two cases of research, we provided validity evidence based on the content, on the response process, and related to the internal structure of the questionnaire, as well as evidence of questionnaire reliability and concurrent validity. As a result of this process, we developed two questionnaires to measure MC in PE lessons that are factorially sound, well-fitting, and reliable instruments. 

Firstly, CONFLICT1-AGE explores the students’ perception of the different possibilities which can cause MC. The most appropriate items were selected according to the theoretical frame of reference [47,48], and confirmed both by the panel of experts and by the students of the focus groups. Evidence related to its content validity, response process validity, internal structure, reliability, and concurrent validity was obtained [63,66,68,69,70,71]. The process ended with a proposal of seven items for the final version of the CONFLICT1-AGE.

The solution or positive transformation of any conflict requires the adoption of a win–win attitude model by the parties involved [51]. For this reason, becoming aware of the inappropriate motor conduct that cause the conflict is the first step to achieving this transformation in the attitude of the participants [47]. This reflection should help the learners involved to know whether their motor conduct have prioritized their personal interests or, on the contrary, have been oriented towards the search for power relations over others. The educational challenge should lead them to orient their motor conduct towards proposals where relationships with others, the enjoyment of the process, and peaceful coexistence play a leading role [7,28,38,49].

Secondly, CONFLICT1-RES examines the students’ perception of the responses elicited by the MC-generating stimulus. The most appropriate items were chosen according to the theoretical framework [47,48], confirmed both by the panel of experts and by the students of the focus groups. Evidence was obtained on its content validity, response process validity, internal structure, reliability, and concurrent validity [63,66,68,69,70,71]. The process ended with a proposal of five items for the final version of CONFLICT1-RES.

Any conflict is a complex process that should be understood in the context of an origin and a reaction. This second questionnaire offers the students enough information to identify their attitude towards the conflict, as receiving and/or generating agents of that relational tension [49,52]. In this case, the reaction to an interpersonal conflict is closely related to the adequate regulation of the negative emotions that this tense relationship triggers [11].

In addition, the combination of both questionnaires will allow PE teachers to quantify the students’ MC level (ICF) in order to propose, design, and put into practice coherent strategies to optimize possible conflictive conducts [49]. Thanks to these questionnaires, the PE teacher will have additional information to decide the transformation of the MCs through the adaptation or modification of the rules of the game (motor game’s internal logic) and/or through pedagogical proposals that facilitate interpersonal dialogue between their students [29,30,49]. These actions could help students to be active actors in the positive transformation of their motor conduct [52]. In short, the instruments proposed could improve these pedagogical strategies and help PE teachers when trying to improve school coexistence from their PE lessons [23,29,48,50,51].

The students’ perception on MC will help PE teachers to complement their observations and data. It will provide PE teachers the possibility of making decisions and designing coherent proposals that improve the education of their students’ motor conduct in favor of creating positive environments that promote school coexistence [4,13,22,23,24], as well as democratic and mutual respect learning [5,6,7,8].

Collectively, the results of this study provide evidence that justifies the functionality of both questionnaires. They focus on the elements that are part of the MC process [49]. The results presented in this article suggest that students can offer their perception about the causes that generate MC, about the conflicting responses derived from the previous stimulus, or even about both actions. These contributions will help PE students and teachers to educate interpersonal relationships, promoting the development of social skills—that is to say, the constructive transformation of conflict situations [23,29,30] and overcoming the difficulties that cause the emergence of interpersonal conflicts [6,25,26,27,28].

## 5. Conclusions

This research is a step forward in understanding the complex phenomenon of conflicts that occur in PE lessons. Educating relational well-being should be a priority in today’s society. 

From a scientific point of view, there is a need for applied research, that is, to promote PE based on scientific evidence. The two tools offered by these two studies could be used in PE lessons and also in other research focused on positive conflict transformation. The psychometric properties of these tools encourage researchers to use them in conjunction with other tools and methodological strategies.

In parallel, these two researches provide a direct and very positive impact on PE teachers. The two questionnaires, CONFLICT1-AGE and CONFLICT1-RES, provide a global vision of the conflict in PE from the students’ point of view. These two questionnaires help to identify the origin, the response, and intensity (ICF) of the MC. Often, teachers consider only their external observations, and this makes it difficult to understand deep interpersonal tensions from the students’ point of view. 

Moreover, the use of these questionnaires implies giving prominence to students’ reflection on their learning in action and helps them to participate in meaningful learning associated with democratic and peaceful interpersonal relationships.

The questionnaires developed in the present study aimed at assessing the core aspects of the MC. However, such an approach prevented the inclusion of the complementary aspects of the construct. Therefore, we encourage future researchers to develop a longer instrument that assesses the complete breadth of the MC construct.

Despite the advantages offered by these questionnaires and their encouraging results, it is important to note that the studies presented must be replicated. The validation of a questionnaire is a continuous process that is always active, and it is necessary that future research uses the CONFLICT1-AGE and the CONFLICT1-RES, relating it to other types of variables to complement the findings shown in this paper.

The evidence provided in our two studies should be confirmed in other groups of participants with different characteristics. This would allow reinforcement the psychometric properties of both questionnaires and their use in different PE contexts.

## Figures and Tables

**Table 1:** Comparison among CFA (Confirmatory factor analysis) measurement models (CONFLICT1-AGE).

Model		χ^2^ (*df*)	*p*	RMSEA (CI 90%)	CFI	TLI
Sample A						
	Original 21-item CONFLICT1-AGE (Version 1)	717.109 (189)	<0.001	0.097 (0.089–0.104)	0.789	0.765
	Deletion of Item 1, Item 2, Item 3, Item 4, Item 8, Item 11, Item 19	409.920 (77)	<0.001	0.120 (0.109–0.132)	0.855	0.829
	Deletion of (a) Item 1, Item 2, Item 3, Item 4, Item 8, Item 11, Item 19; and (b) Item 12 and Item 15	180.781 (54)	<0.001	0.089 (0.075–0.103)	0.927	0.911
	Deletion of (a) Item 1, Item 2, Item 3, Item 4, Item 8, Item 11, Item 19; Item 12 and Item 15; and (c) Item 5 and Item 6	127.820 (35)	<0.001	0.094 (0.077–0.112)	0.941	0.924
	Deletion of (a) Item 1, Item 2, Item 3, Item 4, Item 8, Item 11, Item 19; Item 12, Item 15; (c) Item 5, Item 6; and (d) Item 9, Item 20 (Version 2)	76.165 (20)	<0.001	0.097 (0.075–0.121)	0.944	0.922
Sample B						
	Version 2	42.855 (20)	0.002	0.062 (0.036–0.088)	0.972	0.960
	Deletion of Item 10 (Version 3)	18.621 (14)	0.180	0.033 (0.000–0.069)	0.994	0.991

**Table 2:** Data distribution and factor loadings of CONFLICT1-AGE (Version 3) for Sample B.

Item	Data Distribution (%)					λ
	1	2	3	4	5	
In the game, the conflicts I have caused have been so because I have cheated and this has hurt me.	**65.8**	12.1	4.7	5.0	12.4	0.420
In the game, the conflicts I have caused have been so because someone has not respected the initial agreements.	**45.3**	15.4	12.8	17.1	9.4	0.699
In the game, the conflicts I have caused have been so because someone has cheated.	44.3	3.4	0.7	0.3	**51.3**	0.844
In the game, the conflicts I have caused have been so because someone has insulted me.	**60.1**	1.3	1.3	1.3	35.9	0.500
In the game, the conflicts I have caused have been so because someone has cheated and has not been sanctioned.	**47.7**	0.7	9.7	3.7	38.3	0.765
In the game, the conflicts I have caused have been so because someone has cheated and this affected me	**45.3**	8.4	11.4	19.8	15.1	0.715
In the game, the conflicts I have caused have been so because someone hit me	**68.1**	1.3	2.3	0.3	27.9	0.603

Note. The most selected category for each item is highlighted in bold. 1 = totally disagree, 2 = disagree, 3 = neither agree nor disagree, 4 = agree, 5 = totally agree. All factor loadings were significant at *p* < 0.001.

**Table 3:** Comparison among CFA measurement models (CONFLICT1-RES).

Model	χ^2^ (*df*)	*p*	RMSEA (CI 90%)	CFI	TLI
Sample A					
Original 6-item CONFLICT1-RES	34.176 (9)	<0.001	0.097 (.064–0.132)	0.974	0.957
Deletion of Item 2 (Version 2)	30.380 (5)	<0.001	0.131 (0.088–0.177)	0.973	0.947
Sample B					
Version 2 ^1^	13.350 (5)	0.020	0.075 (0.027–0.125)	0.986	0.972

Note. ^1^ In Version 2 no items were deleted based on the analyses conducted with Sample B.

**Table 4:** Data distribution and factor loadings of CONFLICT1-RES (Version 3) for Sample B.

Item	Data Distribution (%)					λ
	1	2	3	4	5	
In the game, when I caused some conflict I have been insulted.	42.3	1.3	0.7	1.7	**54.0**	0.881
In the game, when someone pushed me to the limit, I have insulted him.	35.9	1.7	2.3	1.7	**58.4**	0.840
In the game, when I caused some conflict, I have been insulted and beaten.	**51.0**	12.8	6.4	12.8	17.1	0.541
In the game, when I caused some conflict, I have been beaten	**69.1**	1.7	0.7	0.0	28.5	0.453
In the game, when someone pushed me to the limit, I have insulted and beaten him.	**54.0**	6.0	3.0	9.7	27.2	0.746

Note. The most selected category for each item is highlighted in bold. 1 = totally disagree, 2 = disagree, 3 = neither agree nor disagree, 4 = agree, 5 = totally agree. All factor loadings were significant at *p* < 0.001.

## Supplementary Materials

The following information is available online at https://www.mdpi.com/1660-4601/17/17/6241/s1, Table S1: Data Distribution of the CONFLICT1-AGE (Version 1), Table S2: Data Distribution of the CONFLICT1-RES (Version 1).
