# Supplementary material for: Development and Validation of Two Questionnaires to Study the Perception of Conflict in Physical Education
Source: Int J Environ Res Public Health. 2020 Aug 27;17(17):6241. doi: 10.3390/ijerph17176241 (PMC7503495; doi:10.3390/ijerph17176241)
Supplement: Supplementary file 1 [file ijerph-17-06241-s001.pdf]

Table S1. Data Distribution of the CONFLICT1-AGE (Version 1).

Item	1	2	3	4	5
In the game the conflicts I caused have been so because I have insulted someone	43.6	0.7	0.3	3.0	**52.3**
In the game the conflicts I caused have been so because of my rules misunderstanding	**76.3**	7.2	6.7	1.2	8.6
In the game, the conflicts I've caused came from me inadvertently bumping into someone else.	34.4	3.2	3.9	9.1	**49.5**
In the game, the conflicts I have caused came from my non-respect of the agreements.	**67.8**	7.4	4.4	0.2	20.3
In the game, the conflicts I have caused have been so because I've hit someone else.	**47.7**	0.7	1.5	3.2	47.0
In the game, the conflicts I have caused have been so because I have cheated.	35.9	3.4	0.3	0.0	**60.4**
In the game, the conflicts I have caused have been so because I have cheated and this has hurt me.	**66.4**	10.1	5.2	5.7	12.6
In the game, the conflicts I have caused have been so because I have made a wrong decision.	**60.9**	1.3	3.4	1.5	32.9
In the game, the conflicts I have caused have been so because I did not respect the game's rule.	**53.2**	8.6	8.4	8.4	21.5
In the game, the conflicts I have caused have been so because I was wrong trying to achieve the objective of the game.	**53.7**	16.9	13.4	2.7	13.3
In the game, the conflicts I have caused have been so because I cheated and have been sanctioned.	**53.9**	0.2	0.2	0.0	45.8
In the game, the conflicts I have caused have been so because someone was wrong in trying to achieve the objective of the game.	**55.5**	8.9	13.6	4.9	17.1
In the game, the conflicts I have caused have been so because someone has not respected the initial agreements.	**47.0**	13.1	12.1	19.0	8.9
In the game, the conflicts I have caused have been so because someone has cheated.	44.1	3.5	1.0	0.2	**51.2**
In the game, the conflicts I have caused have been so because someone has made a wrong decision in the game.	**42.1**	16.9	18.5	14.9	7.6
In the game, the conflicts I have caused have been so because someone has insulted me.	**58.2**	0.8	1.0	1.5	38.4
In the game, the conflicts I have caused have been so because someone has cheated and has not been sanctioned.	**50.7**	0.5	10.4	3.2	35.2
In the game, the conflicts I have caused have been so because someone has cheated and this affected me.	**46.5**	9.9	10.4	17.1	16.1
In the game, the conflicts I have caused have been so because someone has inadvertently bumped into me.	**43.1**	13.4	15.4	16.1	11.9
In the game, the conflicts I have caused have been so because someone did not follow the rules of the game.	**38.8**	11.1	11.7	16.3	22.1
In the game, the conflicts I have caused have been so because someone hit me.	**67.8**	0.8	2.0	0.7	28.7

Note. $n = 596$. No missing values were observed. The most selected category for each item is highlighted in bold. 1 = totally disagree, 2 = disagree, 3 = neither agree nor disagree, 4 = agree, 5 = totally agree.

Table S2. Data Distribution of the CONFLICT1-RES (Version 1).

Item	1	2	3	4	5
In the game, when I caused some conflict I have been insulted.	38,1	1,5	0,8	1,7	**57,9**
In the game, when someone pushed me to the limit, I have beaten him.	**77,2**	1,3	0,8	,7	20,0
In the game, when someone pushed me to the limit, I have insulted him.	34,2	1,3	2,2	1,5	**60,7**
In the game, when I caused some conflict, I have been insulted and beaten.	**50,3**	12,8	8,1	12,4	16,4
In the game, when I caused some conflict, I have been beaten.	**65,6**	1,5	0,7	,2	32,0
In the game, when someone pushed me to the limit, I have insulted and beaten him.	**52,5**	5,0	2,9	12,2	27,3

Note. n = 596. No missing values were observed. The most selected category for each item is highlighted in bold. 1 = totally disagree, 2 = disagree, 3 = neither agree nor disagree, 4 = agree, 5 = totally agree.
